# Humanization: Improving patient and family experience in a public pediatric hospital

**DOI:** 10.1016/j.clinsp.2023.100187

**Published:** 2023-04-02

**Authors:** Jussara de Oliveira Zimmermann, Anna-Dulce S.C. Sampaio, Aide Mitie Kudo, Magda Carneiro-Sampaio

**Affiliations:** Instituto da Criança e do Adolescente (ICr), Hospital das Clínicas da Faculdade de Medicina da Universidade de São Paulo (HCFMUSP), São Paulo, SP, Brazil

In addition to offering advanced diagnostic procedures and treatments for patients with complex, severe, and rare diseases, another equally relevant concern of our Children's Hospital (ICr-HCFMUSP) is the warm welcome, as well as providing the least traumatic experience possible for both inpatients and outpatients and their families. The commitment to reducing the suffering of sick children has always been intrinsic to pediatric practice worldwide. In our Institution, the establishment of formal practices linked to the Humanization of care and the discussion about them date back to the early 1990s, under the coordination of social worker Ms. Maria José Paro Forte and Prof. Yassuhiko Okay. Play therapy has always been part of the care routine and since the 1980´s Occupational Therapists are primarily responsible for these activities and use playing as a strategy for their intervention.

Humanization, established as federal policy in Brazil in 2003, aims at improving the experience of all those involved in health processes ‒ patients, families, employees, managers, and volunteers ‒ in institutions linked to the National Health System (SUS - Sistema Único de Saúde), and it represents a valuable framework for practices and institutional organization.^1^ In the following paragraphs, some ICr-HCFMUSP initiatives and strategies are briefly described to make health care for infants, children, and adolescents more humanized, focusing mostly on hospitalized patients.

A pioneer program called “Child-friendly Diagnosis” (in Portuguese “Diagnóstico Amigo da Criança”) has been developed since 2012 with the involvement of physicians, nurses, and other professionals. Its main objectives have been: i) Reducing the volume of blood withdrawal for laboratory analyses; ii) Reducing exposure to ionizing radiation (X-Ray, particularly for computed tomography), prioritizing ultrasound whenever possible, iii) Reducing the child's emotional suffering during diagnostic procedures, and for this, ICr-HCFMUSP has promoted improvement of ambiance and reduction and even abolition of fasting for blood analyses,^2^^,^^3^ Therapeutic education for chronic diseases aimed at adolescents and families has been another innovative initiative proposed and developed by pediatricians together with other professionals.^4^

Initiatives to improve the ambiance have been transforming the Hospital's atmosphere. The presence of colorful and fun images on the walls and ceiling of some rooms has favored relaxation, joy, and well-being, with a significant impact on patients and families, as well as on the staff. The first intervention began in 2014 and has been represented by the posting of several artwork reproductions by the painter Gustavo Rosa (São Paulo, 1946‒2013) on the walls of various areas of the Hospital, such as the main entrance, outpatient clinics, day hospital, hemodialysis, and emergency room (Figs. 1, 2). Our outpatient clinic, which has 26 posters in its corridors, now looks like a small art gallery, and patients enjoy taking different photos at each visit. More recently, improvements in ambiance were carried out in the Diagnostic Center, with the adaptation of MRI (magnetic resonance imaging) equipment to simulate a yellow submarine at the bottom of a blue ocean full of fish and other animals, while the walls of the blood withdrawal area were completely covered with figures of animals inside a forest (Figs. 3, 4). Children get curious and delighted when entering these spaces and, therefore, are less anxious about the procedures. A virtual reality program is being developed with the aim of reducing the need to anesthetize older children undergoing MRIs.

**Figure 1:**
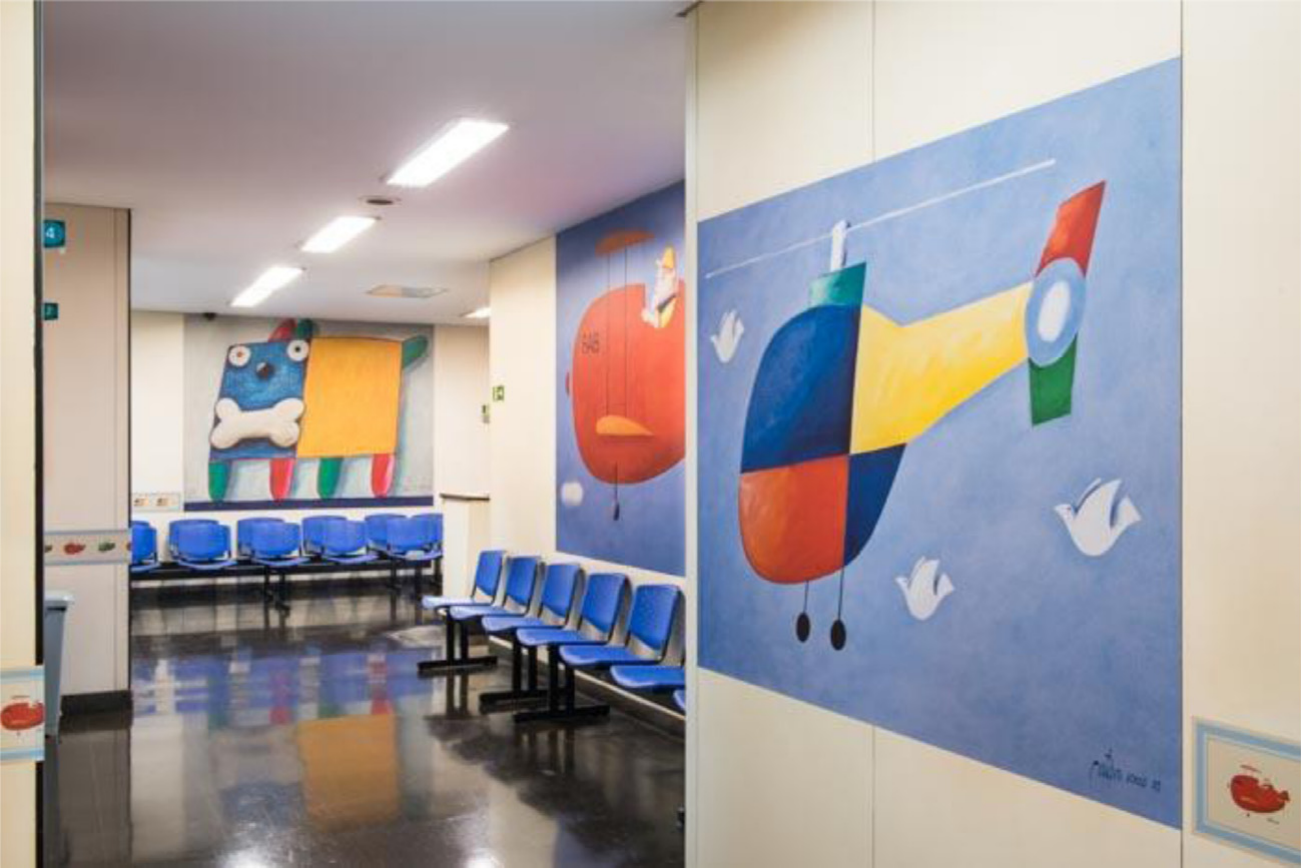
Aspect of an outpatient waiting room.

**Figure 2:**
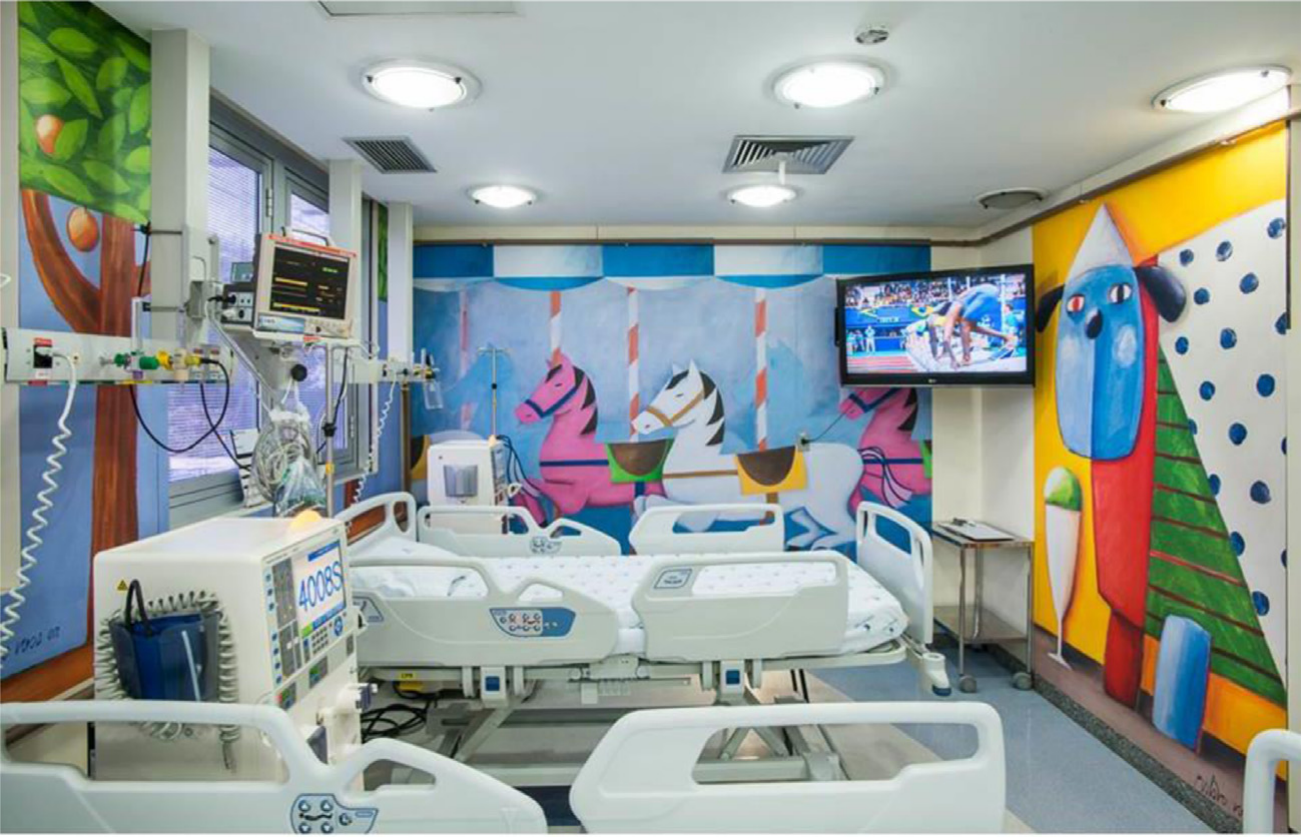
Aspect of the hemodialysis unit.

**Figure 3:**
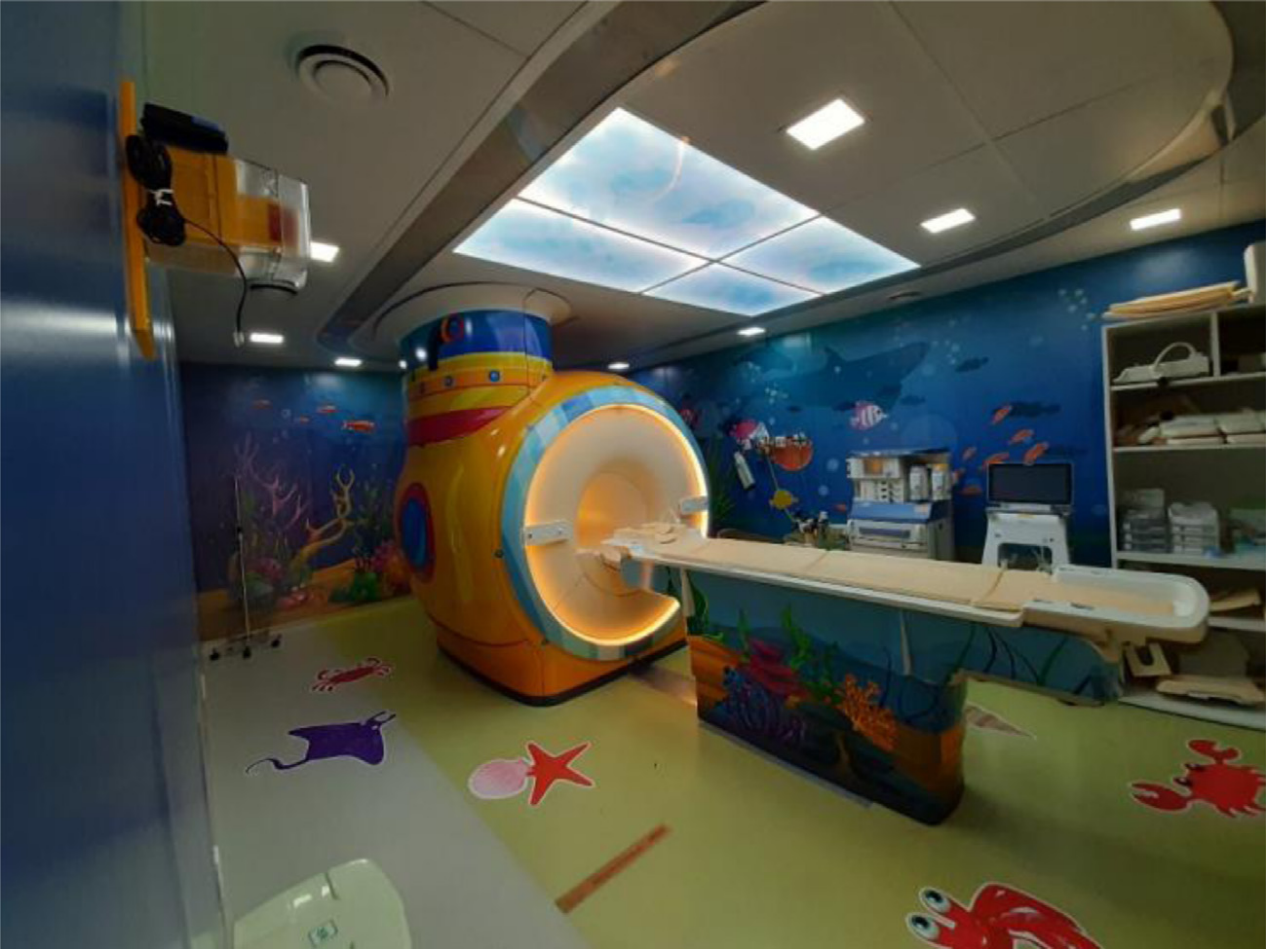
Magnetic Resonance Imaging equipment.

**Figure 4:**
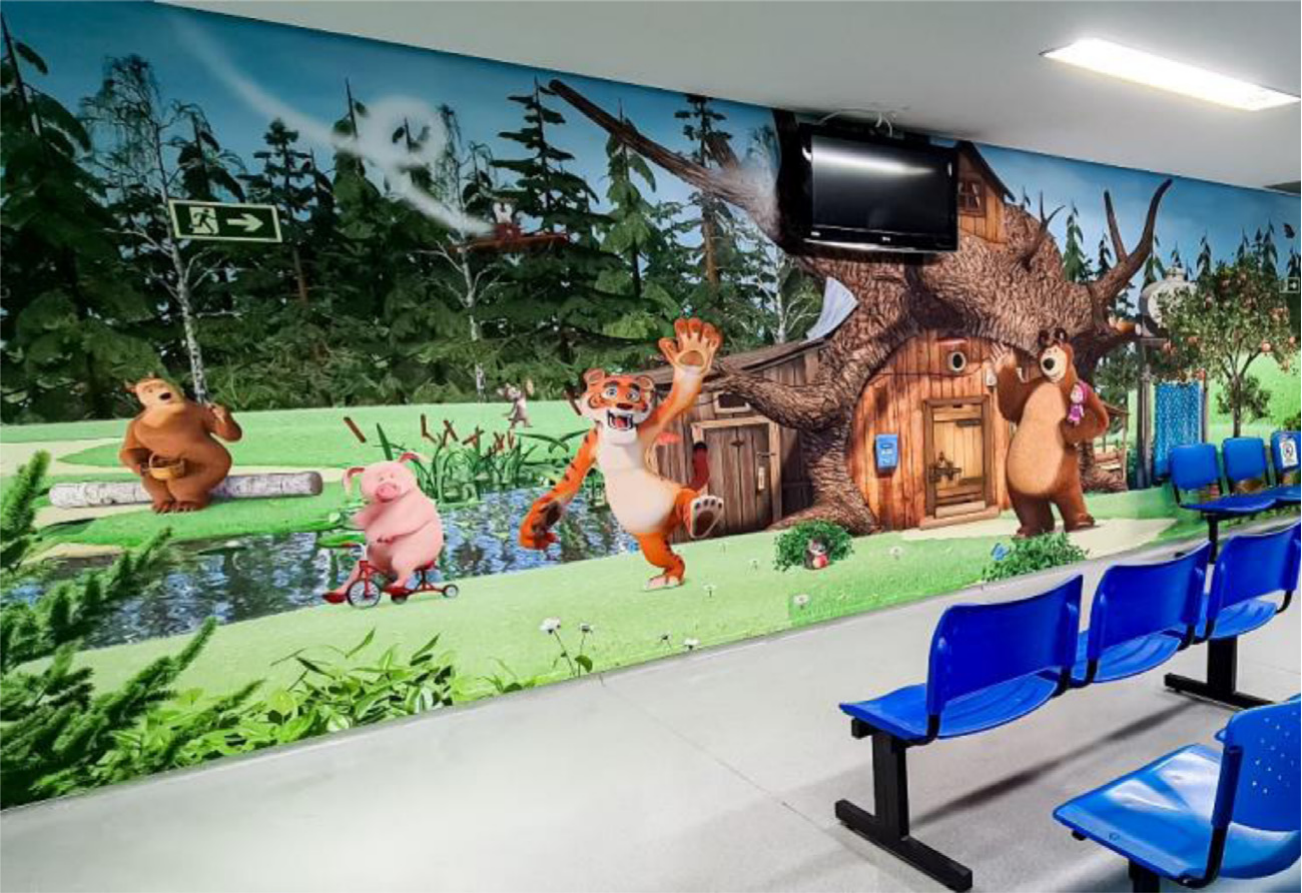
Aspect of the blood withdrawal area.

Playing is an activity inherent to children's daily lives, essential for neuropsychomotor development and for the establishment of social and affective relationships, and guaranteed in the hospital environment. In Brazil, there is a public policy providing for the mandatory existence of toy libraries in pediatric health services, which should represent a privileged space for allowing children to experience pleasant moments of relaxation, learning, social interaction, and stimuli to recover their health.^5^ The use of interactive games and toys is also a resource to help patients in coping with the limitations resulting from their illnesses and hospitalization, with the ultimate objective of improving their quality of life, especially for children with chronic diseases. Special toys and play activities have already been developed in ICr-HCFMUSP with the aim of elucidating diseases and procedures for patients.

Promoting free or therapeutic artistic activities, such as visual arts, videos, and music, to hospitalized and ambulatory patients has been demonstrated as an effective way to humanize the treatment experience.^6^ People with chronic and severe illnesses spend a significant part of their lives in the hospital environment, sometimes their entire lives. Thus, experiences related to education, interpersonal relationships, art, spirituality, and other aspects of life have to happen within the hospital environment, or they will not happen at all.

However, artistic insertion in a hospital environment presents many challenges and limitations to be overcome, especially in tertiary institutions, where strict control of nosocomial infections is crucial. Thus, materials traditionally used in art therapy, such as clay, wood, and stone, are not allowed for hospitalized patients. Even music therapy faces some barriers, as many instruments are made of wood and should only be used by the therapist and always following the Infection Control Committee recommendations. Wind instruments should be avoided. On the other hand, plastic, or metal musical instruments, as well as toys in general, represent better options for patients, as they can be adequately sanitized.

Hospital schooling, adopted as a public policy in Brazil, offers educational content equivalent to that of public schools.^7^ It is largely available at ICr-HCFMUSP and represents an instrument for social inclusion that can mitigate the profound impact of long-lasting or repeated hospitalizations on the education of pediatric patients, compromising their school performance and professional future.

To provide the aforementioned activities to its approximately 200 hospitalized patients and some recreational activities for approximately 250 outpatients per day, the ICr-FMUSP has the collaboration of approximately 400 volunteers from different professional backgrounds. They are organized into groups to offer recreational, educational, and artistic activities (professional musicians and clowns), pet visits, spirituality, and different types of crafts with accompanying parents, among others.

Disseminating the concepts and practices of humanization of care is imperative among all health and administrative professionals for the success of the programs. In addition to a warm welcome, some topics should be addressed, such as cultural, religious, ethnic, gender, and sexuality diversity, non-violent communication, and others.

Based on the principle that “the patient is the most important person of the Institution”, ICr-HCFMUSP has already achieved good welcoming practices and respect for the individuality of patients and their families, as well as among staff members, who need to feel comfortable in their care activities. Moreover, the authors are convinced that more than talks and courses, the dissemination of everyday examples of solidarity, empathy, compassion, and kindness allows the incorporation of good humanization practices aimed at improving the patient's experience in the hospital.

## Conflicts of interest

The authors declare no conflicts of interest.
